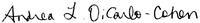# Letter to the Editor

**DOI:** 10.1002/acm2.13963

**Published:** 2023-03-17

**Authors:** Andrea Lynn DiCarlo‐Cohen

**Affiliations:** ^1^ Associate Director for Radiation Countermeasures Research and Emergency Preparedness Radiation and Nuclear Countermeasures Program (RNCP) Division of Allergy Immunology, and Transplantation (DAIT) National Institute of Allergy and Infectious Diseases (NIAID) National Institutes of Health (NIH)

Dear Editors,

My name is Andrea DiCarlo‐Cohen, and I lead the Radiation and Nuclear Countermeasures Program (RNCP), administered by the Division of Allergy, Immunology, and Transplantation (DAIT), National Institute of Allergy and Infectious Diseases (NIAID), within the National Institutes of Health. The RNCP provides funding to researchers to develop medical countermeasures to address gaps in diagnostics and treatments for injuries sustained during a radiological or nuclear public health emergency. I am writing to correct several inaccuracies that appeared in your December 2022 journal issue, in a paper authored by Williams JP et al.^1^ In the manuscript, the authors make incorrect statements about the NIAID RNCP. The specific statements of concern from the publication are outlined and addressed below:

- FROM THE TEXT—*“Unfortunately, despite the subsequent relevant events in Fukushima*, ***federal funding in this area has declined significantly from its initial levels*** *”*
  - RNCP REBUTTAL—It is not correct to say that federal funding in this area has declined significantly from initial levels. When the RNCP was established in 2004, funding via a congressional appropriation to the NIH Office of the Director was set at $45.93 million per year. As of the writing of this letter, the RNCP Fiscal Year 2023 budget is $52.64 million, all of which is earmarked for high dose radiation research.
- FROM THE TEXT—*Acknowledging the loss of expertise within the field, several large projects funded by NASA have included aspects of training as a part of their research program*, ***as did the National Institute of Allergy and Infectious Diseases/Biomedical Advanced Research and Development Authority, Centers for Medical Countermeasures against Radiation program in its initial 5 years of funding***.
  - RNCP REBUTTAL 1 – It is a mischaracterization to state that the Biomedical Advanced Research and Development Authority (BARDA; established in 2006, a year after the 2005 initial award of the Centers) is a part of the National Institute of Allergy and Infectious Diseases’ Centers for Medical Countermeasures against Radiation (CMCR) program. The CMCR program, currently in its 4th iteration, continues to be managed by the NIAID/RNCP. Although BARDA historically provided limited financial support for small Pilot Projects of interest awarded by the consortium, these contributions represent only a very small percentage of the NIAID's overall investment of >$375 M into the CMCR program from 2005 through 2022. NIAID maintains a strong and collegial trans‐government partnership with BARDA, a component of the Administration for Strategic Preparedness and Response, in the Department of Health and Human Services, but the RNCP/NIAID portfolio of grant, cooperative agreement, contract, and inter‐agency awardees is not directly tied to BARDA.
  - RNCP REBUTTAL 2 – Education and training has continued to be a supported aspect within the CMCR program, beyond the initial 5 years of funding. For example, subsequent NIH CMCR funding opportunity announcements RFA‐AI‐19‐012^2^, and RFA‐AI‐14‐055^3^ stated that NIAID would support *“training and education materials in radiobiology research,”* and urged applicants to include plans to establish*“…web‐based training materials, dedicated to the scientific community, in: 1) the principles of radiobiology; 2) use of assays, methods, reagents, animal models, or technologies to study radiobiology; and 3) the regulatory process required for the licensure of new products and technologies.”* In response to that mandate, the CMCR Web‐Based Radiobiology Textbook^4^, which contains 32 chapters written by subject matter experts in radiation biology, was launched in 2018, and continues to serve as a valuable education tool for the radiation research community. In addition, NIAID's investment in radiation sciences has extended beyond the CMCR flagship program (which currently represents only just under 20% of the RNCP Fiscal Year 2023 program budget), to encompass training and education programs supported through other NIAID funding mechanisms, such as Diversity Administrative Supplements (PA‐20‐222^5^) and Research Career Development (K series^6^) awards.

Thank you for the opportunity to address the inaccuracies made in the above‐referenced publication.